# Tissue-type Plasminogen Activator (tPA) Modulates the Postsynaptic Response of Cerebral Cortical Neurons to the Presynaptic Release of Glutamate

**DOI:** 10.3389/fnmol.2016.00121

**Published:** 2016-11-09

**Authors:** Valerie Jeanneret, Fang Wu, Paola Merino, Enrique Torre, Ariel Diaz, Lihong Cheng, Manuel Yepes

**Affiliations:** ^1^Department of Neurology and Center for Neurodegenerative Disease, Emory University School of MedicineAtlanta, GA, USA; ^2^Department of Neurology, Veterans Affairs Medical CenterAtlanta, GA, USA

**Keywords:** tissue-type plasminogen activator (tPA), plasminogen, Ca^2+^/calmodulin-dependent protein kinase II (CaMKII), homeostatic plasticity, protein phosphatase 1

## Abstract

Tissue-type plasminogen activator (tPA) is a serine proteinase released by the presynaptic terminal of cerebral cortical neurons following membrane depolarization (Echeverry et al., 2010). Recent studies indicate that the release of tPA triggers the synaptic vesicle cycle and promotes the exocytosis (Wu et al., 2015) and endocytic retrieval (Yepes et al., 2016) of glutamate-containing synaptic vesicles. Here we used electron microscopy, proteomics, quantitative phosphoproteomics, biochemical analyses with extracts of the postsynaptic density (PSD), and an animal model of cerebral ischemia with mice overexpressing neuronal tPA to study whether the presynaptic release of tPA also has an effect on the postsynaptic terminal. We found that tPA has a bidirectional effect on the composition of the PSD of cerebral cortical neurons that is independent of the generation of plasmin and the presynaptic release of glutamate, but depends on the baseline level of neuronal activity and the extracellular concentrations of calcium (Ca^2+^). Accordingly, in neurons that are either inactive or incubated with low Ca^2+^ concentrations tPA induces phosphorylation and accumulation in the PSD of the Ca^2+^/calmodulin-dependent protein kinase IIα (pCaMKIIα), followed by pCaMKIIα-mediated phosphorylation and synaptic recruitment of GluR1-containing α-amino-3-hydroxy-5-methyl-4-isoxazolepropionic acid (AMPA) receptors. In contrast, in neurons with previously increased baseline levels of pCaMKIIα in the PSD due to neuronal depolarization *in vivo* or incubation with high concentrations of either Ca^2+^ or glutamate *in vitro*, tPA induces pCaMKIIα and pGluR1 dephosphorylation and their subsequent removal from the PSD. We found that these effects of tPA are mediated by synaptic N-methyl-D-aspartate (NMDA) receptors and cyclin-dependent kinase 5 (Cdk5)-induced phosphorylation of the protein phosphatase 1 (PP1) at T320. Our data indicate that by regulating the pCaMKIIα/PP1 balance in the PSD tPA acts as a homeostatic regulator of the postsynaptic response of cerebral cortical neurons to the presynaptic release of glutamate.

## Introduction

Tissue-type plasminogen activator (tPA) is a serine proteinase with pleotropic roles in the central nervous system (CNS). Accordingly, tPA released from endothelial cells into the intravascular space has a fibrinolytic effect mediated by its ability to catalyze the conversion of plasminogen into plasmin (Camiolo et al., 1971), and tPA released from glia modulates the permeability of the blood-brain barrier (BBB; Yepes et al., 2003), induces neuroglial coupling (An et al., 2014) and activates proinflammatory cell signaling pathways (Zhang et al., 2007). Intriguingly, the rapid release of neuronal tPA following membrane depolarization also has a robust effect on synaptic function. In line with these observations, a growing body of experimental evidence indicates that tPA mediates the development of neuronal plasticity in *in vitro* and *in vivo* models of long-term potentiation (Qian et al., 1993), visual cortex plasticity (Müller and Griesinger, 1998), learning (Seeds et al., 1995, 2003) and stress-induced anxiety (Pawlak et al., 2003).

Recent studies indicate that one of the mechanisms whereby tPA induces synaptic plasticity is by regulating the composition of the active zone and promoting the exocytosis (Wu et al., 2015) and subsequent endocytic retrieval (Yepes et al., 2016) of glutamate-containing synaptic vesicles from the presynaptic terminal of cerebral cortical neurons. However, although these effects are pivotal for the development of synaptic plasticity, the sustained activation of the synaptic vesicle cycle may also cause an excitotoxic injury to the postsynaptic terminal, which would suggest a neurotoxic effect for tPA in the CNS (Wang et al., 1998). Yet, these observations are in sharp contrast with a growing body of experimental evidence indicating that tPA has a neuroprotective effect in the brain mediated by its ability to activate cell signaling pathways that are pivotal for the detection and adaptation to metabolic stress (Echeverry et al., 2010; Haile et al., 2012; Wu et al., 2012, 2013a; Yepes, 2015).

The postsynaptic density (PSD) is a highly dynamic electron-dense structure anchored to the inner surface of the postsynaptic terminal that contains protein complexes that regulate the postsynaptic response to the presynaptic release of neurotransmitters. The composition and size of the PSD change rapidly in response to variations in synaptic activity. For example, cerebral ischemia, glutamate treatment, and membrane depolarization (Aronowski and Grotta, 1996; Martone et al., 1999; Dosemeci et al., 2001) cause a rapid increase in its thickness associated with phosphorylation and accumulation of the alpha subunit of the Ca^2+^/calmodulin-dependent protein kinase II (CaMKIIα).

CaMKIIα is a serine/threonine kinase that regulates the postsynaptic response of excitatory glutamatergic synapses to increase in the concentration of Ca^2+^ (Shakiryanova et al., 2011). Under resting conditions, substrate access to the CaMKIIα binding site is blocked by the autoinhibitory pseudosubstrate segment of the protein. However, the influx of Ca^2+^ leads to Ca^2+^/calmodulin-dependent phosphorylation of CaMKIIα at T286 (pCaMKIIα), relieving its autoinhibition and rendering the kinase active even after Ca^2+^ is removed (Hell, 2014). CaMKIIα phosphorylation has a direct effect on synaptic activity under physiological and pathological conditions (Lisman et al., 2012; Skelding et al., 2012). For example, glutamatergic synapses that are “silent” because they harbor N-methyl-D-aspartate (NMDA) but not α-amino-3-hydroxy-5-methyl-4-isoxazolepropionic acid (AMPA) receptors, become active following pCaMKIIα-induced phosphorylation and recruitment of GluR1-containing AMPA receptors to the PSD (Liao et al., 2001).

Several serine/threonine protein phosphatases (PP) can dephosphorylate and thus regulate pCaMKIIα activity. However, although protein phosphatase 1 (PP1), PP2A and PP2C can all dephosphorylate pCaMKIIα (Strack et al., 1997), each PP seems to act in a specific cellular compartment. Hence, while pCaMKIIα is targeted selectively in the cytosol by PP2A, in the PSD pCaMKIIα is primarily dephosphorylated by PP1 (Shields et al., 1985). PP1 activity is regulated by its interaction with a group of inhibitory proteins that includes the inhibitor-1 (I-1; Endo et al., 1996), the I-1 homolog DARPP-32, and inhibitor-2 (I-2; Huang et al., 1999). Protein kinase A (PKA)-dependent I-1 phosphorylation at T35 stabilizes the PP1-I-1 complex, thereby suppressing PP1 activity. Conversely, PP1 is activated by calcineurin-mediated I-1 dephosphorylation at T35 (Colbran, 2004). Despite the relevance of these observations, recent experimental evidence has uncovered calcineurin- and I-1-independent mechanisms for the regulation of neuronal PP1 activity. For example, cyclin-dependent kinase 5 (Cdk5)-induced PP1 phosphorylation at T320 inhibits PP1 by blocking access to its substrates (Goldberg et al., 1995); importantly, several studies indicate that this mechanism of PP1 regulation is pivotal for the control of the G2/M phase of the cell cycle (Kwon et al., 1997) and the development of synaptic plasticity (Hou et al., 2013).

The work presented here indicates that the presynaptic release of tPA has a bidirectional effect on the composition and function of the postsynaptic terminal of glutamatergic synapses of cerebral cortical neurons that depends on the baseline levels of neuronal activity and the extracellular concentrations of Ca^2+^. Hence, in neurons with low baseline levels of CaMKIIα tPA induces its NMDAR-mediated phosphorylation at T286 (pCaMKIIα) and subsequent accumulation in the PSD. These events are followed by pCaMKIIα-induced phosphorylation and recruitment of GluR1-containing AMPARs to the PSD. In contrast, in neurons with high baseline levels of pCaMKIIα and pGluR1-containing AMPARs in the PSD due to neuronal depolarization *in vivo* or incubation with high concentrations of Ca^2+^ or glutamate *in vitro*, tPA induces their dephosphorylation and subsequent removal to extrasynaptic sites. We found that this homeostatic effect is mediated by tPA’s ability to regulate the Cdk5-dependent phosphorylation of PP1 at T320. Based on these data, here we propose a model in which the presynaptic release of tPA is a homeostatic mechanism that modulates the response of the postsynaptic terminal to the presynaptic release of glutamate.

## Materials and Methods

### Animals and Reagents

Strains were 8–12 weeks-old male wild-type (Wt) and T4 mice (with a 20-fold increase in tPA expression in neurons (Madani et al., 1999), kindly provided by Professor J. D. Vassalli and Dr. R. Mandani, University of Geneva, Switzerland) on a C57BL/6J background. Experiments were approved by the Institutional Animal Care and Use Committee of Emory University, Atlanta GA, following guidelines established by ARRIVE (Animal Research: reporting *in vivo* Experiments). Recombinant murine tPA and proteolytically inactive tPA (itPA) with an alanine for serine substitution at the active site Ser481 (S481A) were acquired from Molecular Innovations (Novi, MI, USA). Other reagents were antibodies against the following proteins: protein phosphatase (I-1) phosphorylated at T35 (Santa Cruz Biotechnologies; Santa Cruz, CA, USA), PP1 phosphorylated at T320 (Cell Signaling; Boston, MA, USA), microtubule-associated protein- 2 and β-actin (MAP-2; Sigma-Aldrich; St. Louis, MO, USA), postsynaptic density protein-95 (PSD-95), and CaMKIIα phosphorylated at T286, total GluR1 and GluR1 phosphorylated at S831 (Abcam, Cambridge, MA, USA). The cell permeable calcium chelator BAPTA-AM, bicuculline, the NMDA receptor blocker MK-801, tetrodotoxin (TTX), and the cell-permeable CaMKII inhibitor KN-62 were purchased from Tocris Bioscience (Minneapolis, MN, USA). Other reagents were glycine, glutamate, and the proteosomal inhibitor MG132 (Sigma-Aldrich, St. Louis, MO, USA), protease and phosphatase inhibitors (Roche, Indianapolis, IN, USA), DABCO mounting media (Fluka, St. Louis, MO, USA) and the Cdk-5 inhibitor roscovitine (Rosc; Calbiochem; Billerica, MA, USA).

### Neuronal Cultures

Cerebral cortical neurons were cultured from E16 to E18 Wt mice as described elsewhere (Wu et al., 2012). Briefly, the cerebral cortex was dissected, transferred into Hanks’ balanced salt solution containing 100 units/ml penicillin, 100 μg/ml streptomycin, and 10 mM HEPES, and incubated in trypsin containing 0.02% DNase at 37°C for 15 min. Then tissue was triturated, and the supernatant was re-suspended in B27-supplemented neurobasal medium containing 2 mM l-glutamine and plated onto 0.1 mg/ml poly-l-lysine-coated wells.

### Isolation of Synaptoneurosomes and PSD-Enriched Fractions

Wt cerebral cortical neurons were treated as described below. The forebrain of T4 mice and their Wt littermate controls was extracted after bilateral common carotid artery occlusion (BCCAO) as described below, scrapped in cold fractionation buffer (1 mM EGTA, 0.25 M sucrose, 25 mM HEPES pH 8.1) in the presence of inhibitors of proteases and phosphatases. Cells and tissue were homogenized using a 5 ml tissue grinder, and the homogenate was centrifuged at 2000 g for 5 min to remove cell debris. The supernatant (S1) was transferred to a new tube and centrifuged at 32,000 g for 10 min. The pellet (P2) containing synaptoneurosomes and used or resuspended in 1 ml of a solution containing 150 mM KCl and 0.5% Triton and centrifuged at 275,000 g for 1 h to extract the PSD. The pellets (P3) were washed again with 0.5 ml of 150 mM KCl and 0.5% Triton buffer, and centrifuged at 275,000 g for 1 h. The PSD-containing pellets (P3) were lysed in 50 mM Tris-HCl/0.3% SDS buffer for protein assay. To test the purity of these preparations, extracts were probed with Western blot analysis with antibodies against PSD-95 (detects the PSD), syntaxin-1 (detects the presynaptic membrane) and synaptophysin (detects synaptic vesicles). Our data indicate that these extracts are highly enriched for PSD-95 and have undetectable levels of both syntaxin-1 and synaptophysin (data not shown).

### Bilateral Common Carotid Artery Occlusion (BCCAO)

Wt and T4 mice were anesthetized with isofluorane and their carotid arteries were exposed and either clipped during 1 min or kept patent (sham operation), as described elsewhere (Echeverry et al., 2010). A second group of animals underwent 5 min of reperfusion followed by either a second episode of 60 s BCCAO or sham-operation. Cerebral perfusion in the forebrain was monitored with a laser Doppler (Perimed), and only animals with >85% decrease in cerebral perfusion with complete reperfusion after BCCAO were included in this study. Immediately after the end of BCCAO, animals were transcardially perfused with cold PBS, brains were harvested and their cerebral cortex was dissected.

### Western Blot Analysis

Wt cerebral cortical neurons (*n* = 4 per group) were incubated with either vehicle (control) or 5 nM of tPA during 1.5, 3 or 5 min; or with 5 nM of proteolytically active or inactive tPA during 5 min in the presence of either 10 μM of MK-801, or 20 μM of BAPTA-AM, or 10 μM of the cell-permeable CaMKII inhibitor KN-62; or with either BAPTA-AM or KN-62 alone; or during 5 min with 200 μM of glycine and 20 μM of bicuculline and 2 μM or 10 μM of glutamate, in the presence of either vehicle (control) or 5 nM of tPA, or pretreated during 30 min or 4 h with either 1 μM of TTX, or 50 μM of Rosc or 10 μM of the proteosome inhibitor MG132, respectively, followed by treatment with 5 nM of tPA. The forebrain of Wt and T4 mice (*n* = 4 animals per group) was harvested immediately after either 1-min of BCCAO or sham-operation, or 1 min of BCCAO followed by 5 min of reperfusion and another minute of either BCCAO or sham-operation. In each case PSD-enriched fractions prepared as described above were homogenized, protein concentration was quantified using the BCA assay, and 2.5 μg were loaded per sample, separated by 4–20% linear gradient polyacrylamide gel, transferred to a PVDF membrane by semi-dry transfer system, blocked with 5% non-fat dry milk in Tris-buffered saline pH 8.0 with 0.1% Tween 20 buffer, and immunoblotted with antibodies against pCaMKIIα (1:1000), total CamKII, (1:5000), PSD-95 (1:1000), β-actin (1:5000), pGluR1 (1:1000), total GluR1 (1:1000), I-1 phosphorylated at T35 (1:1000) or PP1 phosphorylated at T320 (1:1000). Each observation was repeated 4–14 times.

### Proteomics and Quantitative Phosphoproteomics

Proteomics and quantitative phosphoproteomics analyses were performed as described elsewhere (Wu et al., 2012; Dammer et al., 2015) in PSD extracts prepared from Wt cerebral cortical neurons treated 60 s with 5 nM of tPA or vehicle (control; *n* = 3 preparations per group). For pathway analysis we used the DAVID Bioinformatics Database. Log_2_ (tPA-treated/control) values of the average protein intensity ratios were centered so that the fit gauss curve midpoint (mean) fell at zero. Log_2_ values 1.96 standard deviations from the mean (changed with 95% confidence, with absolute value greater than 0.709) were considered as changing and these protein identities and quantifications were considered in the analysis that followed.

### Immunogold Electron Microscopy Studies

Brains from Wt and T4 mice (*n* = 4) were fixed by transcardial perfusion with 4% PFA in 0.1 M phosphate buffer (pH 7.2) for 20 min followed by immersion-fixation overnight at 4°C. Monolayers of Wt cerebral cortical neurons (*n* = 3 preparations) were fixed with 4% PFA in 0.1 M PBS overnight at 4°C. Brains were harvested, sectioned onto 50 μm cuts and permeabilized in 0.05% Triton X-100 for 10 min. Cells were permeabilized with 0.1 μg/ml digitonin. After permeabilization, brain sections and cells were incubated in PBS containing 5% rabbit serum, 5% BSA, and 0.1% gelatin to block potential non-specific interaction between immunoreagents and samples. After several washes brain sections and monolayer cells were fixed with 2.5% glutaraldehyde in 0.1 M PB. Silver enhancement using Aurion R-gen SE-EM kit was then conducted following manufacturer’s instructions. Brain sections and monolayer cells were then fixed with 0.5% osmium tetroxide for 15 min, dehydrated and embedded in Eponate 12 resin. Then areas of the frontal cortex or cell monolayers were dissected out from flat embedded vibrating microtome sections and re-embedded, cut onto 70 nm sections, stained with uranyl acetate and lead citrate, and examined with a JEOL JEM-1400 transmission electron microscope (Tokyo, Japan) equipped with a Gatan US100 CCD camera (Pleasenton, CA, USA). The thickness of the PSD was measured as described elsewhere (Dosemeci et al., 2001). Briefly, images were magnified 4× in ImageJ and the PSD was outlined by hand. The average thickness of the PSD was calculated by dividing the area of the outlined PSD by the length of the postsynaptic membrane. Each observation was repeated in three different cultures and 120 observations were performed per culture, and in 4 Wt and T4 mice (50 neurons were examined per animal at Bregma: 0.5 mm, lateral: 1 mm and ventral: 1 mm (Paxinos and Franklin, 2001) in each mouse.

### Immunohistochemistry and Quantification of pCaMKIIα, pGluR1 and Total GluR1

Wt cerebral cortical neurons were incubated during 90 s in HBSS-containing 2.5 mM of calcium alone or in the presence of 5 nM of proteolytically active or inactive tPA. A second group of cells was incubated during 90 s with 5 nM of active or proteolytically inactive tPA in the presence of HBSS containing 0–1.8 mM of calcium. A third group of cells were incubated during 90 s or 5 min in the presence of 5 nM of tPA or vehicle (control) with HBSS media containing 2.5 mM of calcium, 200 μM of glycine, 20 μM of bicuculline and 1 μM of glutamate. The brains of Wt and T4 mice (*n* = 4 per group) were harvested immediately after 60 s of BCCAO or sham-operation, fixed and cut onto 20 μM sections at Bregma: 0.5 mm, lateral: 1 mm and ventral: 1 mm (Paxinos and Franklin, 2001) cells were fixed for 15 min in 4% paraformaldehyde containing 4% sucrose, permeabilized for 5 min with 50 ug/ml digitonin prepared in Tris buffer saline (TBS), blocked for 30 min in 0.1% casein, 5% goat or donkey serum and double labeled with antibodies against CaMKIIα phosphorylated at T286 (1:2500) and MAP-2 (1:20,000), or antibodies against GluR1 phosphorylated at S831 and PSD-95, and counterstained with phalloydin. A sub-group group of cells was not permeabilized and stained with antibodies against total GluR1. Brain cuts were stained with antibodies against pCaMKIIα (1:10,000) and heat maps were generated in photoshop. Coverslips were washed and mounted using Polyvinyl-alcohol/DABCO mounting media. To quantify pCaMKIIα, pGluR1 and GluR1-positive puncta, 60× pictures taken from dendrites in each experimental group were electronically magnified 4× and the number of either pCaMKIIα, or pGluR1, or GluR1-positive puncta was quantified with the cell counter of ImageJ.

### Statistical Analysis

Statistical analysis was performed with two-tailed *t* test and two-way analysis of variance (ANOVA) with Greenhouse-Geisser correction, as appropriate. *p*-values of <0.05 were consider as significant.

## Results

### TPA Increases the Thickness of the Postsynaptic Density of Cerebral Cortical Neurons

**Figure 1:**
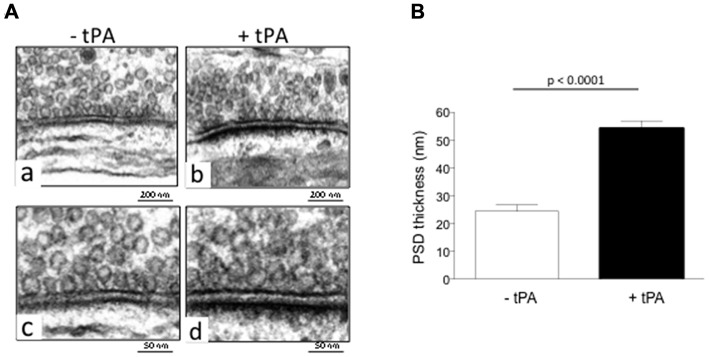
**Type plasminogen activator (tPA) increases the thickness of the postsynaptic density (PSD). (A)** Representative electron microscopy micrographs from the PSD of wild-type (Wt) cerebral cortical neurons incubated during 60 s with either vehicle (control; **a,c**) or 5 nM of tPA **(b,d)**. Magnification 15,000× in **(a,b)** and 30,000× in **(c,d)**. **(B)** Mean thickness of the PSD of 360 Wt cerebral cortical neurons per group incubated during 60 s with either vehicle (control; white bar) or 5 nM of tPA (black bar). Lines denote SEM.

Our earlier work indicates that tPA activates the synaptic vesicle cycle in glutamatergic cerebral cortical neurons (Wu et al., 2015). To investigate whether tPA also has an effect on the postsynaptic terminal, we used electron microscopy to measure the thickness of the PSD of Wt cerebral cortical neurons incubated during 60 s with either 5 nM of tPA or a comparable volume of vehicle (control). We chose this dose because our previous studies indicate that it increases the concentration of tPA in the synaptic cleft to values near to those most likely found after the induction of membrane depolarization (Haile et al., 2012). We found that the thickness of the PSD increases from 24.44 ± 2.31 nm in vehicle (control)-treated neurons to 54.21 ± 2.71 nm in cells incubated with tPA (*n* = 360; *p* < 0.0001; Figure 1).

### TPA Induces CaMKIIα Phosphorylation

Because protein recruitment to the PSD leads to an increase in its thickness (Dosemeci et al., 2001), then we decided to use liquid chromatography coupled to tandem mass spectrometry (LC-MS/MS) to study changes in protein abundance in synaptoneurosomes prepared from Wt cerebral cortical neurons treated during 60 s with 5 nM of tPA or a comparable volume of vehicle (control). Remarkably, these studies showed that compared to vehicle (control)-treated neurons tPA induces a 160% increase in the abundance of CaMKIIα in the synapse (*p* < 0.009). Because CaMKIIα translocates from the cytosol to the PSD following its phosphorylation at T286 (pCaMKIIα), and since the recruitment of pCaMKIIα has been associated with increase in the thickness of the PSD during synaptic activity (Dosemeci et al., 2001; Hell, 2014), then we decided to use immobilized metal affinity chromatography (IMAC) followed by LC-MS/MS to quantify the abundance of phosphorylated proteins in synaptoneurosomes prepared from Wt cerebral cortical neurons treated during 60 s with 5 nM of tPA or a comparable volume of vehicle (control). We found that compared to controls, synaptoneurosomes from neurons treated with tPA have a 194% increase in the abundance of pCaMKIIα.

**Figure 2:**
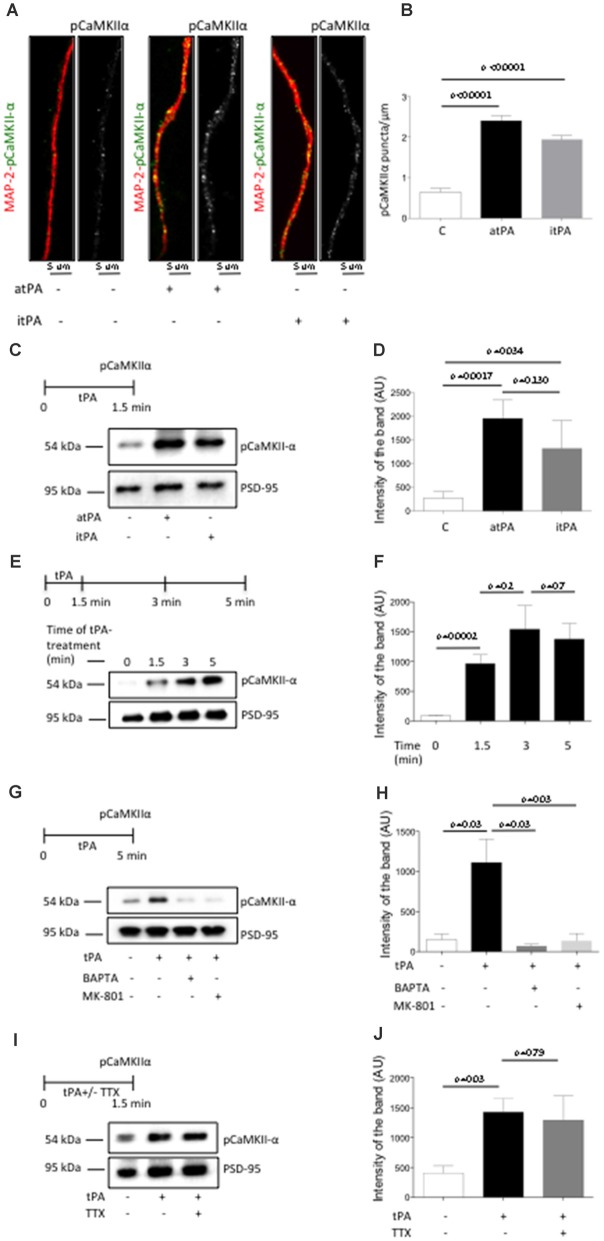
**TPA induces Ca^2+^/calmodulin-dependent protein kinase II (CaMKIIα) phosphorylation and accumulation in the PSD. (A)** Representative immunocytochemical staining for microtubule-associated protein-2 (MAP-2; red) and CaMKIIα phosphorylated at T286 (pCaMKIIα; green and white) in dendrites from Wt cerebral cortical neurons incubated 90 s with vehicle (control) or 5 nM of proteolytically active or inactive tPA (atPA and itPA, respectively). Magnification 100×. **(B)** Mean number of pCaMKIIα-positive puncta in 275 dendrites from Wt cerebral cortical neurons incubated 90 s with either vehicle (control; white bar), or atPA (black bar), or itPA (gray bar). Lines denote SEM. **(C,D)** Experimental design and representative Western blot analysis **(C)** and quantification of the mean intensity of the band **(D)** for pCaMKIIα expression in extracts prepared from the PSD of Wt cerebral cortical neurons incubated 90 s with vehicle control, or 5 nM of either atPA or itPA. Lines denote SD; *n* = 4. **(E,F)** Experimental design and representative Western blot analysis **(E)** and quantification of the mean intensity of the band **(F)** for pCaMKIIα expression in extracts from the PSD of Wt cerebral cortical neurons incubated 0–5 min with 5 nM of tPA. **(G,H)** Experimental design and representative Western blot analysis **(G)** and quantification of the mean intensity of the band **(H)** for pCaMKIIα expression in PSD extracts prepared from Wt cerebral cortical neurons incubated 5 min with 5 nM of tPA, alone or in the presence of either 10 μM of MK-801 or 50 μM of BAPTA-AM. *n* = 4; lines denote SD. **(I,J)** Experimental design and representative Western blot analysis **(I)** and quantification of the mean intensity of the band **(J)** for pCaMKIIα expression in PSD extracts prepared from Wt cerebral cortical neurons incubated with 1 μM of tetrodotoxin (TTX) followed by 5 min of treatment with 5 nM of tPA. *n* = 4; lines denote SD.

Our data indicate that tPA increases the abundance of pCaMKIIα in the synapse. Then, to determine whether this effect occurs in the postsynaptic terminal, we quantified the number of pCaMKIIα-positive puncta in 275 dendrites from Wt cerebral cortical neurons treated during 60 s with 5 nM of either proteolytically active or inactive tPA, or with a comparable volume of vehicle (control). Our data indicate that tPA increases the expression of pCaMKIIα in dendrites of Wt cerebral cortical neurons, and that this effect does not require the conversion of plasminogen into plasmin (Figures 2A,B). Despite the fact that these experiments show that tPA increases the expression of pCaMKIIα in the postsynaptic terminal, they do not indicate that this necessarily happens in the PSD. Thus, we decided to study the expression of pCaMKIIα in PSD extracts prepared from Wt cerebral cortical neurons treated with 5 nM of proteolytically active or inactive tPA. We found that tPA increases the expression of pCaMKIIα in the PSD by a mechanism that does not require the generation of plasmin (Figures 2C,D), that this effect is maximal after 5 min of incubation (Figures 2E,F) and that it requires the influx of Ca^2+^ via NMDA receptors (Figures 2G,H). Importantly, our loading controls with PSD-95 indicate that in each experimental condition we probed equal concentrations of the PSD and that therefore the observed effect of tPA on pCaMKIIα expression is not an epiphenomenon caused by the increase in the thickness of the PSD.

Our earlier work indicates that tPA induces the presynaptic release of glutamate (Wu et al., 2015). Then it is conceivable to postulate that the increase in pCaMKIIα abundance observed in the PSD following treatment with tPA is caused by glutamate released from the presynaptic terminal in response to tPA treatment instead of a direct effect of tPA on the postsynaptic terminal. To test this possibility we studied the expression of pCaMKIIα in PSD extracts prepared from Wt cerebral cortical neurons in which glutamatergic neurotransmission was abrogated by incubation with TTX before treatment with tPA. Our data indicate that the effect of tPA on pCaMKIIα expression in the PSD is independent of the effect of tPA on the presynaptic release of glutamate (Figures 2I,J).

**Figure 3:**
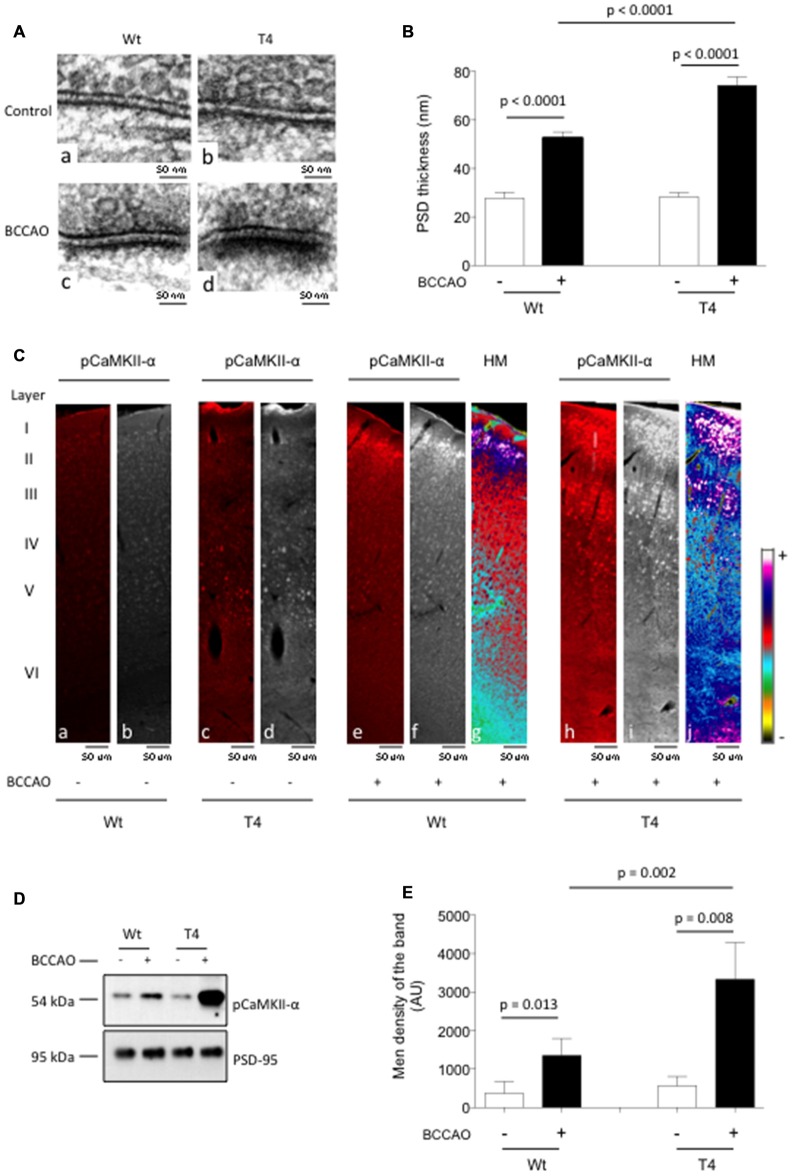
***In vivo* effect of the release of neuronal tPA on CaMKIIα phosphorylation and accumulation in the PSD. (A)** Representative electron microscopy micrographs of the PSD of neurons from the frontal cortex of Wt **(a,c)** and T4 **(b,d)** mice following 1 min of either sham operation **(a,b)** or bilateral common carotid artery occlusion (BCCAO; **c,d**). Magnification: 30,000×. **(B)** Mean thickness of 200 PSDs per experimental group from neurons from the frontal cortex of Wt and T4 mice 60 s after either sham operation (white bars) or BCCAO (black bars). Lines denote SEM. **(C)** Representative micrographs **(a–f,h,i)** from the frontal cortex of Wt and T4 mice stained with antibodies against pCaMKIIα (red in **a,c,e,h** white in **d,f,i**) 60 s after either sham operation or BCCAO. Panels **(g,j)** correspond to heat maps (HT) from panels **(f,i)** Magnification: 40×. **(D,E)** Representative Western blot analysis **(D)** and mean intensity of the band **(E)** for pCaMKIIα expression in extracts from the forebrain of Wt and T4 mice 60 s after either sham operation or BCCAO. Lines in **(E)** depict SD; *n* = 4.

In previous studies we showed that 1 min of BCCAO induces the rapid release of neuronal tPA without causing cell death (Echeverry et al., 2010). Then, to study the *in vivo* significance of our *in vitro* findings we quantified the thickness of the PSD and the expression of pCaMKIIα in the cerebral cortex of mice overexpressing neuronal tPA (T4 mice, with a 20-fold increase in the release of neuronal tPA following membrane depolarization) and their Wt littermate controls following 1 min of either BCCAO or sham-operation. Our data indicate that BCCAO increases the thickness of the PSD from 27.87 ± 2.40 nm to 52.58 ± 2.55 nm in Wt mice (*p* < 0.0001; *n* = 200), and from 28.38 ± 1.66 nm to 74.29 ± 3.55 nm in T4 animals (*p* < 0.0001; *n* = 200; Figures 3A,B). Furthermore, our immunohistochemical studies revealed that BCCAO induces a marked increase in pCaMKIIα expression in the II, IV and V cortical layers of Wt animals (Figure 3C, panels b and f) and that this effect is significantly enhanced by the overexpression of neuronal tPA (Figure 3C, panels d and i). To determine whether the release of neuronal tPA induces the phosphorylation and translocation of CaMKIIα to the PSD *in vivo*, we studied the expression of pCaMKIIα in PSD extracts prepared from the forebrain of Wt and T4 mice following 1 min of either sham operation or BCCAO. Our results show that the release of neuronal tPA induced by BCCAO induces the phosphorylation and recruitment of pCaMKIIα to the PSD *in vivo* and that this effect is significantly enhanced by neuronal overexpression of tPA (Figures 3D,E). As stated above for our *in vitro* experiments, the use of equal concentrations of PSD extracts per experimental condition ensures that this is not an epiphenomenon caused by the observed increase in the thickness of the PSD, but instead that it is due to a direct effect of neuronal tPA on CaMKIIα phosphorylation and its subsequent recruitment to the PSD.

### TPA has a Bidirectional Effect on CaMKIIα Phosphorylation

**Figure 4:**
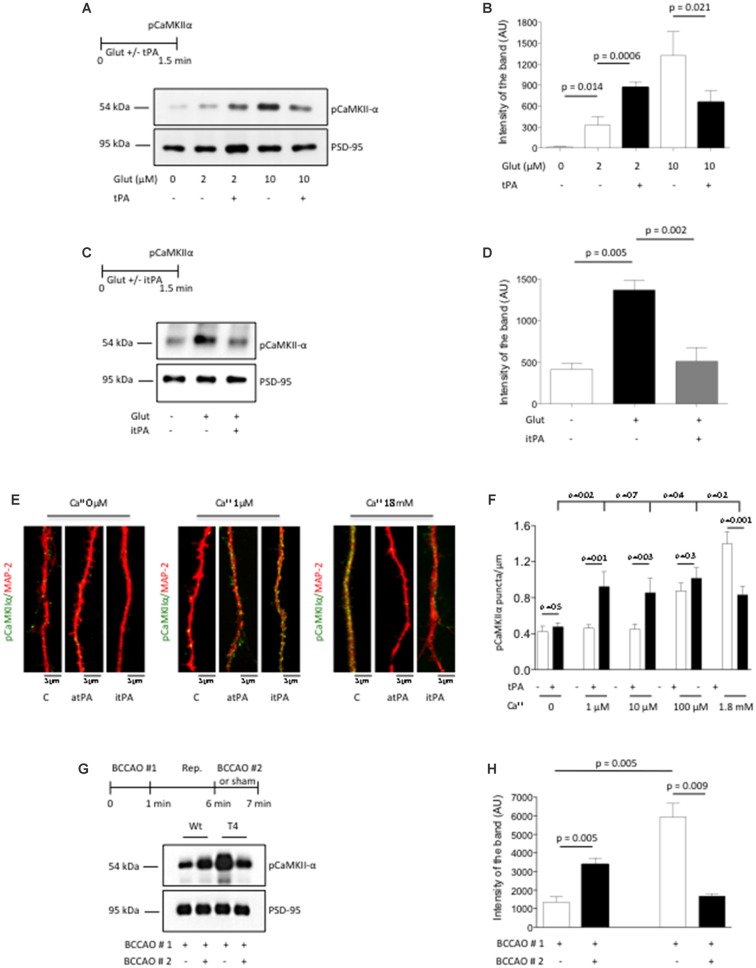
**TPA has a bidirectional effect on pCaMKIIα expression in the post-synaptic density. (A,B)** Experimental design and representative Western blot analysis **(A)** and quantification of the mean intensity of the band **(B)** for pCaMKIIα expression in extracts from the PSD from Wt cerebral cortical neurons incubated with 200 μM of glycine, 20 μM of bicuculline and either 2 μM or 10 μM of glutamate, in the presence of 5 nM of tPA or a comparable volume of vehicle (control). Lines denote SD; *n* = 4. **(C,D)** Experimental design and representative Western blot analysis **(C)** and quantification of the mean intensity of the band **(D)** for pCaMKIIα expression in extracts from Wt cerebral cortical neurons incubated with vehicle (control), or a combination of 10 μM of glutamate, glycine and bicuculline either alone or in the presence of 5 nM of itPA. Lines denote SD; *n* = 4.** (E)** Representative micrographs of pCaMKIIα expression in dendrites from Wt cerebral cortical neurons following 90 s of treatment with either 5 nM of tPA or vehicle (control) in the presence of 0–1.8 mM of calcium. Magnification: 100×. **(F)** Mean number of pCaMKIIα-positive puncta in dendrites from Wt neurons incubated with 5 nM of tPA or vehicle (control) in the presence of 0–1.8 mM of calcium. Bars denote SEM. *n* = 350–375 dendrites per experimental condition. **(G,H)** Experimental design and representative Western blot analysis **(E)** and quantification of the mean intensity of the band **(F)** for pCaMKIIα expression in PSD extracts prepared from the forebrain of Wt and T4 mice following 60 s of (BCCAO #1) followed by 5 min of reperfusion (Rep.) and 60 s of either a second (BCCAO #2) or sham operation. Lines denote SD; *n* = 4.

Our *in vitro* and *in vivo* data indicate that the release of neuronal tPA promotes the phosphorylation and subsequent recruitment of pCaMKIIα to the PSD of cerebral cortical neurons that have low baseline levels of pCaMKIIα. To investigate the effect of tPA on the phosphorylation and recruitment of pCaMKIIα to the PSD of neurons with previously existing high baseline levels of pCaMKIIα, we studied the effect of tPA on the expression of pCaMKIIα in PSD extracts from Wt cerebral cortical neurons in which the baseline levels of pCaMKIIα were previously increased by incubation with a combination of 200 μM of glycine and 20 μM of bicuculline, and either low (2 μM) or high (10 μM) concentrations of glutamate. We found that glutamate induces a dose-dependent increase in the expression of pCaMKIIα in the PSD of cerebral cortical neurons. However, while tPA potentiates the effect of 2 μM of glutamate, it attenuates the effect of 10 μM of glutamate on the phosphorylation and recruitment of pCaMKIIα to the PSD (Figures 4A,B), by a mechanism that does not require the conversion of plasminogen into plasmin (Figures 4C,D).

Because the extracellular concentrations of Ca^2+^ increase during neuronal activity, and since our data indicate that tPA-induced CaMKIIα phosphorylation and accumulation in the PSD requires the influx of Ca^2+^ via NMDA receptors (Figures 2G,H), we decided to study whether variations in the extracellular concentrations of Ca^2+^ have an effect on the direction of the effect of tPA on CaMKIIα phosphorylation. To accomplish this goal we quantified the number of pCaMKIIα-positive puncta in dendrites of 375 neurons incubated with 0–1.8 mM of Ca^2+^ in the presence of either 5 nM of tPA or vehicle (control). We found that tPA fails to induce CaMKIIα phosphorylation in absence of Ca^2+^. However, while pCaMKIIα expression did not change in the presence of either 1 μM or 10 μM of Ca^2+^ alone, it increased in the presence of 5 nM of tPA in combination with either one of these concentrations of Ca^2+^. In contrast, pCaMKIIα expression increased following incubation with 100 μM or 1.8 mM of Ca^++^ alone. However, while tPA did not change the effect of 100 μM of Ca^2+^on pCaMKIIα expression, it significantly attenuated it in cells incubated with 1.8 mM (Figures 4E,F). Importantly, regardless of the Ca^2+^ concentrations in each experimental group we did not observe a statistically significant difference between the different tPA-treated groups, suggesting that tPA brings pCaMKIIα expression from lower or higher baseline levels to a preset homeostatic physiological level.

To confirm the *in vivo* relevance of these findings we performed similar observations with PSD extracts prepared from the forebrain of Wt and T4 mice exposed to 60 s of (BCCAO # 1- first release of neuronal tPA) followed by 5 min of reperfusion and 60 s of either sham-operation or a second (BCCAO # 2- second release of neuronal tPA). We found that the effect of BCCAO on CaMKIIα phosphorylation and its subsequent recruitment to the PSD is significantly enhanced by neuronal overexpression of tPA. However, while an additional episode of neuronal release of tPA induced by a second BCCAO further increased the levels of pCaMKIIα in the PSD of Wt mice, it had the opposite effect on T4 animals in which the first episode of BCCAO had already induced very high levels of pCaMKIIα in the PSD (Figures 4G,H). Together, our data show that tPA has a bidirectional effect on the phosphorylation and recruitment of CaMKIIα to the PSD that depends on the baseline levels of pCaMKIIα.

### TPA Regulates the Activation of Protein Phosphatase-1 in the Post-Synaptic Density

**Figure 5:**
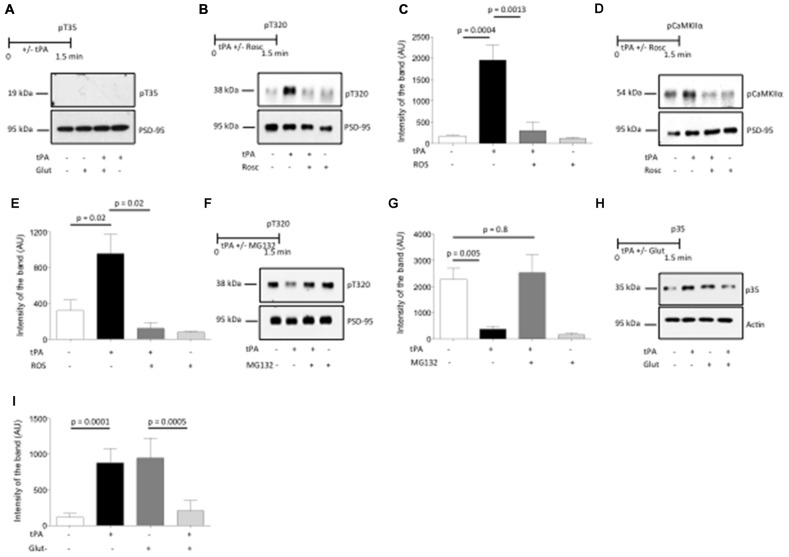
**Cyclin-dependent kinase 5 (Cdk5) mediates the effect of tPA on CaMKIIα phosphorylation and its subsequent accumulation in the PSD. (A)** Experimental design and representative Western blot analysis of the expression of inhibitor 1 phosphorylated at T35 in PSD extracts prepared from Wt cerebral cortical neurons incubated with vehicle (control), or a combination of glutamate, glycine and bicuculline either alone, or in the presence of 5 nM of tPA, or with tPA alone. **(B,C)** Experimental design and representative Western blot analysis **(A)** and quantification of the mean intensity of the band **(B)** for the expression of protein phosphatase-1 (PP1) phosphorylated at T320 in PSD extracts from Wt cerebral cortical neurons incubated with 5 nM of tPA alone or in the presence of 50 μM of the Cdk5 inhibitor roscovitine (Rosc); *n* = 4. Lines denote SD. **(D,E)** Experimental design and representative Western blot analysis **(C)** and quantification of the mean intensity of the band **(D)** for the expression of pCaMKIIα in PSD extracts prepared from Wt cerebral cortical neurons treated with tPA in the presence or absence of the Cdk5 inhibitor Rosc; *n* = 4. Lines denote SD. **(F,G)** Experimental design and representative Western blot analysis **(E)** and quantification of the mean intensity of the band **(F)** for the expression PP1 phosphorylated at T320 in PSD extracts from Wt cerebral cortical neurons incubated with 5 nM of tPA alone or in the presence of 10 μM of the proteasomal inhibitor MG132; *n* = 4. Lines denote SD. **(H,I)** Experimental design and representative Western blot analysis **(G)**, and quantification of the mean intensity of the band **(H)** for the expression of the Cdk5 activator p35 in PSD extracts prepared from Wt cerebral cortical neurons either left untreated or incubated with a combination of glutamate, bicuculline and glycine alone or in the presence of 5 nM of tPA, or incubated with tPA alone; *n* = 4. Lines denote SD.

Dephosphorylation by PP1 regulates pCaMKIIα activity in the PSD (Bradshaw et al., 2003). Thus, we postulated that the effects of tPA on CaMKIIα phosphorylation are mediated by changes in the phosphorylation status of PP1. Because phosphorylation or dephosphorylation of (I-1) at T35 (pT35I-1) causes PP1 inactivation or activation, respectively, we decided to study the expression of pT35I-1 in PSD extracts prepared from Wt cerebral cortical neurons incubated with vehicle (control), or with a combination of glutamate, bicuculline and glycine, either alone or in combination with 5 nM of tPA, or with tPA alone. Our data show that in cerebral cortical neurons tPA does not have an effect I-1 phosphorylation at T35 (Figure 5A).

Recent studies have found that Cdk-5-induced phosphorylation of PP1 at T320 (pPP1) is a calcineurin-independent mechanism for PP1 inactivation that plays a central role in the development of synaptic plasticity (Hou et al., 2013). Thus, to investigate whether PP1 inactivation by its phosphorylation at T320 mediates tPA-induced CaMKIIα phosphorylation and recruitment to the PSD in neurons with low baseline levels of pCaMKIIα, we studied the expression of pPP1 (T320) in PSD extracts prepared from Wt cerebral cortical neurons treated with 5 nM of tPA or vehicle (control) in the presence or absence of the Cdk5 inhibitor Rosc. We found that tPA induces PP1 phosphorylation at T320 and that this effect is abrogated by Cdk5 inhibition (Figures 5B,C). To further determine whether Cdk5-induced PP1 phosphorylation at T320 mediates the effect of tPA on CaMKIIα phosphorylation, we studied the expression of pCaMKIIα in extracts prepared from the PSD of Wt cerebral cortical neurons treated with tPA in the presence or absence of Rosc. Together, our data indicate that Cdk5 activation mediates the effect of tPA on PP1 inactivation by phosphorylation at T320, and CaMKIIα phosphorylation and its subsequent recruitment to the PSD (Figures 5D,E).

Then we investigated if PP1 activation by its dephosphorylation at T320 mediates tPA-induced pCaMKIIα dephosphorylation and its ensuing removal from the PSD in neurons with previously existing high baseline levels of pCaMKIIα, and whether Cdk-5 mediates this effect. Because Cdk-5 inactivation is caused by ubiquitin-mediated degradation of its activator p35 (Patrick et al., 1998), we studied the expression of PP1 phosphorylated at T320 in cells incubated with glycine, bicucculine and 10 μM of glutamate (to increase baseline pCaMKIIα expression) and tPA, in the presence or absence of the proteasomal inhibitor MG132. We found that tPA induces PP1 dephosphorylation at T320 in neurons with high baseline levels of phosphorylated PP1, and that this effect is abrogated by proteasomal inhibition (Figures 5F,G).

Our data show that the bidirectional effect of tPA on CaMKIIα phosphorylation and recruitment to the PSD is mediated by Cdk5 activation or inactivation. Then we postulated that this effect is mediated by a bidirectional effect of tPA on the expression of the Cdk5 activator p35. To test this hypothesis, we studied the expression of p35 in plasma membrane extracts from synaptoneurosomes prepared from Wt cerebral cortical neurons either left untreated (to maintain low levels of baseline neuronal activity) or incubated with a combination of glycine, bicucculine and glutamate (to increase baseline levels of neuronal activity), followed by treatment with 5 nM of tPA. Our data indicate that tPA increases the expression of p35 in previously inactive synapses, while it decreases p35 expression in synapses that are already active (Figures 5H,I).

### TPA Induces pCaMKIIα-Dependent Recruitment of GluR1-Containing AMPA Receptors to the PSD

**Figure 6:**
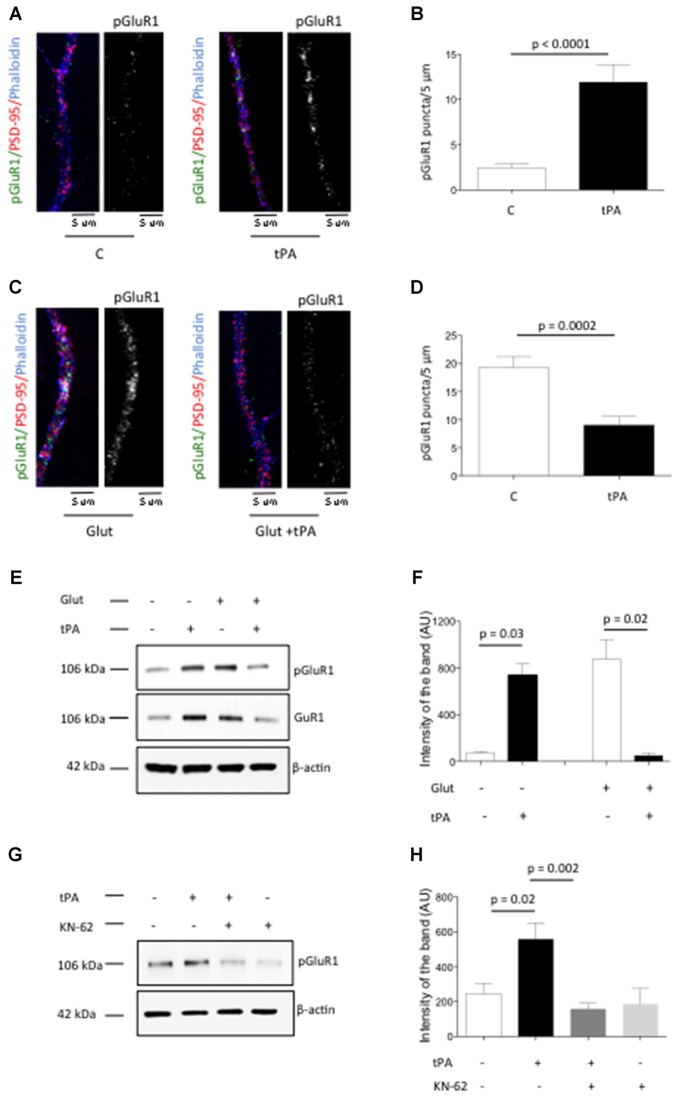
**Effect of tPA on the phosphorylation and recruitment of GluR1-containing α-amino-3-hydroxy-5-methyl-4-isoxazolepropionic acid (AMPA) receptors to the PSD. (A)** Representative micrographs of immunocytochemistry studies with antibodies against GluR1 phosphorylated at S831 (green and white), PSD-95 (red) and phalloydin (blue) in dendrites from Wt neurons treated during 90 s with either vehicle (control) or 5 nM of tPA. Magnification: 100×. **(B)** Mean number of pGluR1-positive puncta in dendrites from 75 neurons treated 90 s with vehicle (control) or 5 nM of tPA. Lines denote SEM. **(C)** Representative micrographs of immunocytochemical staining for GluR1 phosphorylated at S831 (green and white), PSD-95 (red) and phalloydin (blue) in dendrites from 50 Wt neurons treated during 90 s with a combination of 200 μM of glycine, 20 μM of bicuculline and 10 μM of glutamate (Glut), in the presence of 5 nM of tPA or a comparable volume of vehicle (control). Magnification: 100×. **(D)** Mean number of pGluR1-positive puncta in the distal dendrites from 50 neurons treated 90 s with a combination of 200 μM of glycine, 20 μM of bicuculine and 10 μM of glutamate (Glut), alone or in the presence of 5 nM of tPA. Lines denote SD. **(E,F)** Representative Western blot analysis for pGluR1 and total GluR1 expression **(E)** and mean intensity of the band **(F)** in PSD extracts prepared from Wt cerebral cortical neurons treated during 90 s with vehicle (control) or 5 nM of tPA, either alone or in the presence of 200 μM of glycine, 20 μM of bicuculine and 10 μM of glutamate (Glut). Lines in **(F)** denote SD. *n* = 4. **(G,H)** Representative Western blot analysis for pGluR1 and total GluR1 expression **(G)** and mean intensity of the band **(H)** in PSD extracts prepared from Wt cerebral cortical neurons treated with vehicle (control) or 5 nM of tPA, either alone or in the presence of 10 μM of the cell-permeable CaMKIIα inhibitor KN-62. Lines in **(H)** denote SD. *n* = 4.

The conversion of “silent” into active synapses requires pCaMKIIα-induced phosphorylation of GluR1 receptors at S831 (pGluR1) and their subsequent recruitment to the PSD (Lee et al., 1998; Liao et al., 2001). Based on these observations we decided to study the expression of pGluR1 in dendrites from Wt cerebral cortical neurons treated with either 5 nM of tPA or a comparable volume of vehicle control. We found that the number of pGluR1-positive puncta/5 μm increases from 2.44 ± 0.43 in vehicle (control)-treated cells to 11.92 ± 1.91 in neurons incubated in the presence of tPA (*p* < 0.0001; Figures 6A,B). To study the effect of tPA on pGluR1 expression in neurons with already increased baseline levels of pGluR1 we performed similar observations in cells incubated with glycine, bicucculine and 10 μM of glutamate (to increase the baseline expression of pGluR1) either alone or in the presence of 5 nM of tPA. We found that the number of pGluR1-positive puncta/5 μm decreases from 19.32 ± 1.97 in neurons treated with glutamate alone to 9 ± 1.59 in cells treated with glutamate in the presence of tPA (*p* = 0.0002; Figures 6C,D). To ensure that the effect of tPA on pGluR1 occurs in the PSD, we studied the expression of pGluR1 and total GluR1 in PSD extracts prepared from Wt cerebral cortical neurons incubated with or without a combination of glycine, bicuculline and glutamate, alone or in the presence of either 5 nM of tPA or a comparable volume of vehicle (control). Our results show that in neurons with low baseline levels of pGluR1 and total GluR1, tPA induces their recruitment and phosphorylation to the PSD. In contrast, when baseline the levels of total and pGluR1 levels are already increased, tPA induces its dephosphorylation and mobilization to extrasynaptic sites (Figures 6E,F). Finally, to determine whether the observed effect of tPA on the phosphorylation and mobilization to the PSD of AMPA receptors-containing GluR1 subunits is mediated by pCaMKIIα, we studied the expression of pGluR1 in PSD extracts from Wt cerebral cortical neurons incubated either with tPA or vehicle (control), alone or in the presence of the cell-permeable CaMKIIα inhibitor KN-62. We found that pCaMKIIα mediates the effect of tPA on GluR1 phosphorylation and its recruitment to the PSD (Figures 6G,H).

## Discussion

A substantial body of experimental evidence indicates that tPA modulates the efficiency of glutamatergic neurotransmission in the CNS (Samson and Medcalf, 2006). Accordingly, tPA is stored in non-glutamatergic dense core secretory granules in the presynaptic terminal of cerebral cortical neurons and its rapid release at extrasynaptic sites following membrane depolarization modulates the exocytosis of glutamate-containing SVs by a sequence of events that include translocation of SVs from the reserve pool to the synaptic release site via Ca^2+^-mediated synapsin I phosphorylation and recruitment of βII-spectrin to the active zone (Wu et al., 2015). Importantly, the effect of tPA on SVs exocytosis does not lead to their depletion from the presynaptic terminal because tPA also promotes their endocytic retrieval from the presynaptic membrane via dynamin-I dephosphorylation (Yepes et al., 2016).

The release of tPA at extrasynaptic sites and its aforementioned effect on the synaptic vesicle cycle suggest that in the CNS tPA plays a neuromodulatory role on glutamatergic neurotransmission. In agreement with these observations, the work presented here indicates that tPA modulates the response of the postsynaptic terminal to the presynaptic release of gutamate via its ability to regulate the pCaMKIIα/PP1 switch in the PSD. Furthermore, our data show that this role of tPA is independent of its ability to induce the presynaptic release of glutamate (Wu et al., 2015) and instead is mediated by postsynaptic NMDA receptors. This effect may be pivotal for the homeostatic preservation of synaptic function and circuit stability during conditions of either synaptic depression or in those that such as cerebral ischemia and seizures are characterized by the excitotoxic release of glutamate.

The PSD is a disk-like structure apposed to the postsynaptic membrane of the dendritic spine and assembled by specialized protein complexes that determine the postsynaptic response to the presynaptic release of neurotransmitters (Okabe, 2007). The PSD is highly dynamic and its thickness changes in response to variations in synaptic activity. For example, an increase in the concentration of glutamate in the synaptic cleft, either pharmacologically added (Dosemeci et al., 2001) or provoked by the induction of cerebral ischemia (Aronowski and Grotta, 1996), causes a rapid and transient increase in the thickness of the PSD. The data presented here show that the release of neuronal tPA or incubation with tPA also increases the thickness of the PSD, suggesting a functional link between glutamatergic neurotransmission and tPA release. Together with our earlier studies indicating that tPA also increases the size of the active zone of the presynaptic terminal (Wu et al., 2015), these observations suggest that tPA induces the morphological and functional changes in the presynaptic and postsynaptic terminals required for successful glutamatergic neurotransmission.

CaMKII is a holoenzyme that accounts for a large proportion of the total protein content of the PSD. Indeed, there are approximately 80 CaMKII complexes per 0.1 μm^2^ of PSD, and in some PSDs there is more CaMKII than the scaffold protein PSD-95 (Chen et al., 2008). In agreement with these observations, the enlargement of the PSD in neurons exposed to either high concentrations of glutamate or to an ischemic injury has been associated with phosphorylation and accumulation of pCaMKIIα in the postsynaptic terminal (Aronowski and Grotta, 1996; Martone et al., 1999; Dosemeci et al., 2001). Our data indicate that the release of neuronal tPA mediates and modulates the effect of glutamate on the phosphorylation and recruitment of pCaMKIIα to the PSD, and that this modulatory role is mediated by Ca^2+^ influx via NMDA receptors. Moreover, it is important to note that our studies with PSD extracts ensure that this effect occurs in the PSD instead of other cellular compartments where CaMKII also plays important roles. Furthermore, our data are in agreement with work by others (Nicole et al., 2001) indicating an interaction between tPA and NMDA receptors. However, in contrast with these reports, in our model this interaction does not require tPA’s proteolytic properties, which is in line with observations by several other groups (Samson et al., 2008) reporting a non-proteolytic interaction between tPA and NMDA receptors that is mediated by the low-density lipoprotein receptor-related protein-1 (LRP-1). The results presented here indicate that the effect of tPA on the phosphorylation and recruitment of CaMKIIα to the PSD require synaptic NMDA receptors. Unfortunately, the experimental design of our work does not allow us to determine whether NMDA receptors also mediate the opposite effect (i.e., decreasing pCaMKIIα in the PSD of already active synapses).

Several studies have suggested that the phosphorylation and accumulation of pCaMKIIα in the PSD is neurotoxic in pathological situations characterized by the excitotoxic release of glutamate (Coultrap et al., 2011; Skelding et al., 2012). Based on these observations it is conceivable to postulate that tPA may have a neurotoxic effect mediated by its ability to promote the phosphorylation and accumulation of pCaMKIIα in the PSD. However, our data indicates that this is not the case. Instead, we found that when pCaMKIIα is already increased in the PSD tPA induces its dephosphorylation via PP1 activation. These results agree with previous studies demonstrating that tPA has a homeostatic effect in the synapse under physiological and pathological conditions (Wu et al., 2012, 2013b; Yepes, 2015). Importantly, our data indicate that as shown by others (Hou et al., 2013) the baseline levels of PP1 phosphorylated at T35 are almost undetectable, and that tPA does not have an effect on I-1 phosphorylation at T35. Instead, our results indicate that tPA has a direct effect on PP1 phosphorylation at T320.

AMPARs are assembled by four subunits with distinct electrophysiological properties (GluR1, 2, 3 and 4). Accordingly, AMPARs containing GluR2 subunits show lower permeability for Ca^2+^ while GluR1 subunits confer high Ca^2+^ permeability. Several electrophysiological studies indicate that a large fraction of glutamatergic synapses contain NMDA but not AMPA receptors. These are considered as “silent synapses” that however can be activated by the recruitment of AMPA receptors to the PSD (Liao et al., 2001). More specifically, during synaptic inactivity GluR1-containing AMPARs are restricted from the synapses, but they are recruited to the PSD by activity-induced synaptic enhancement (Hayashi et al., 2000). pCaMKIIα plays a pivotal role in the conversion from silent to active synapses. Indeed, pCaMKIIα-induced phosphorylation of GluR1 subunits at S831 (pGluR1) is necessary for their recruitment to the PSD and the resultant enhancement in AMPAR conductance required for the activation of the postsynaptic terminal (Kristensen et al., 2011). Importantly, depending on the baseline level of neuronal function, activation of NMDA receptors is capable to induce both, phosphorylation and dephosphorylation of GluR1 subunits, with the subsequent activation or inhibition of synaptic activity (Lisman and Zhabotinsky, 2001). This event, known as bidirectional synaptic plasticity (Lee et al., 2000), plays a pivotal role not only in learning but also in the development of homeostatic plasticity needed to maintain neuronal activity within a physiological range (Turrigiano, 2008). The data presented here show that tPA mediates the development of this process and that its direction depends on the extracellular concentrations of Ca^2+^ and the baseline level of neuronal activity.

It has been proposed that a highly sensitive CaMKIIα/PP-1 switch regulates the response of the postsynaptic terminal to changes in the extracellular concentrations of Ca^2+^ (Bradshaw et al., 2003). However, the biochemical mechanisms that determine the direction of this switch are still unknown. We found that an as yet uncharacterized interaction between tPA and Cdk5 regulates the CaMKIIα/PP1 balance in the PSD according to the baseline levels of pCaMKIIα phosphorylation and the extracellular concentrations of Ca^2+^, and that this effect leads to mobilization of GluR1-containing AMPA receptors in and out of the PSD with the subsequent formation of active or silent synapses, respectively.

**Figure 7:**
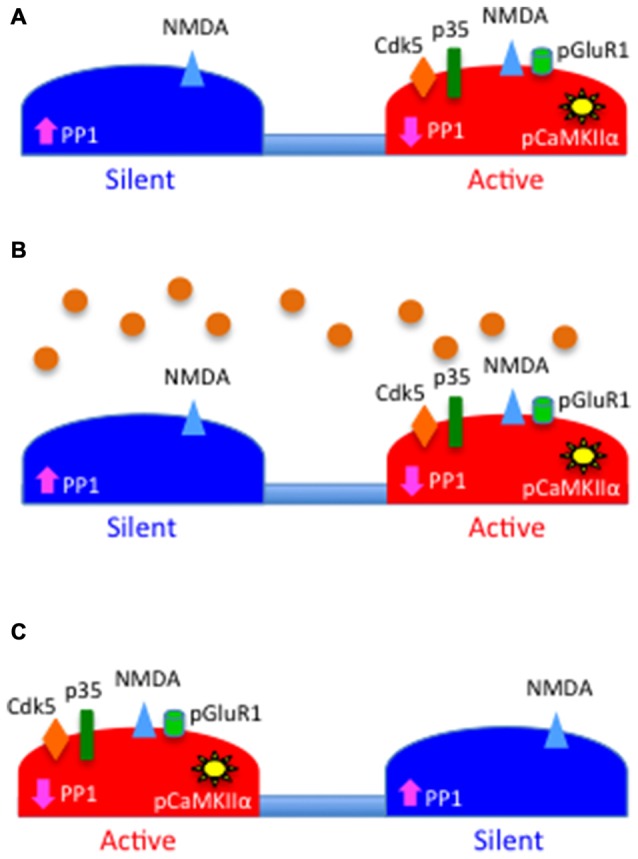
**Schematic representation of the proposed role for tPA as a homeostatic regulator of synaptic function. (A)** PSD of a silent (blue) and an active (red) synapse. Note that the levels of PP1 activity are high in the silent synapse and low in the active synapse owning to the absence or presence, respectively, of Cdk5-induced PP1 phosphorylation at T320, which leads to correspondingly low and high levels of pCaMKIIα. The figure also depicts the presence of N-methyl-D-aspartate (NMDA; blue triangles) but not GluR1-containing AMPA receptors (green cylinders) in the silent synapse. **(B)** Synaptic depolarization induces the release of tPA (orange circles) that then interacts with the PSD of both synapses. **(C)** tPA induces the recruitment of p35 to the PSD of the silent synapse and its proteasomal degradation in the active synapse. This leads in the silent synapse to Cdk5-induced inactivation of PP1 by phosphorylation at T320, with a resultant increase in pCaMKIIα in the PSD, and pCaMKIIα-induced phosphorylation and recruitment of GluR1 subunits to the PSD.

In summary, based on the data presented here we postulate a model whereby its ability to have a bidirectional effect on the CaMKIIα/PP-1 switch and the subsequent phosphorylation and recruitment of GluR1-containing AMPA receptors to the PSD, tPA is a homeostatic regulator of the postsynaptic response to the presynaptic release of glutamate (Figure 7). We postulate that this homeostatic mechanism is pivotal for the stabilization of individual synapses that are either depressed by lack of synaptic input or overstimulated by an excitotoxic injury.

## Author Contributions

VJ, FW, PM, AD, ET and LC: performed the experiments; MY: designed the experiments and wrote the manuscript.

## Funding

This work has been supported in part by National Institutes of Health Grants NS-079331 (to MY) and NS-091201 (to MY).

## Conflict of Interest Statement

The authors declare that the research was conducted in the absence of any commercial or financial relationships that could be construed as a potential conflict of interest.
